# Neglected Patellar Tendon Rupture Treated With a Single Semitendinosus Tendon in One-Stage Reconstruction Surgery: A Case Report of an Unusual Injury Mechanism

**DOI:** 10.7759/cureus.80699

**Published:** 2025-03-17

**Authors:** Petar Vukman, Marko Kadija, Svetlana Sreckovic, Miljan Bilanovic, Darko Milovanovic

**Affiliations:** 1 Orthopedics and Traumatology, University Clinical Centre of Serbia, Belgrade, SRB; 2 Faculty of Medicine, University of Belgrade, Belgrade, SRB; 3 Anesthesiology and Perioperative Medicine, University Clinical Centre of Serbia, Belgrade, SRB; 4 Orthopedics and Traumatology, University Hospital Medical Center Bezanijska Kosa, Belgrade, SRB

**Keywords:** gunshot wound, neglected injury, patellar tendon reconstruction, patellar tendon rupture, semitendinosus tendon graft, single stage reconstruction

## Abstract

We present the case of a 23-year-old patient who sustained a gunshot wound (GSW) during a mass shooting and had an unrecognized rupture of the patellar tendon. The patient was treated with a single-stage reconstruction, using only the semitendinosus tendon with preserved distal tendon insertion and two tunnels, transtibial and trans patellar, along with McLaughlin augmentation of the repair. The reconstruction proved strong enough to withstand the postoperative rehabilitation process. At the one-year follow-up, the patient walked without pain, had a full range of motion, and had enough muscle strength in the upper leg. The MRI showed the injured leg had almost the same Insall-Salvati ratio and Caton-Deschamps index as the uninjured leg.

## Introduction

Patellar tendon ruptures occur 0.48 to 1.09 times per 100,000 person-years, making them quite rare [1,2]. Men in their third and fourth decades of life account for the majority of these injuries, with sports-related trauma being the most common mechanism of injury where abrupt quadriceps contraction during knee flexion leads to tendon rupture [3]. Injury of the knee’s extensor mechanism compromises all the functions necessary for daily activities, such as extending the knee when standing or walking. Siwek and Rao classified these injuries as acute, conducting treatment within two weeks after the injury, or chronic, conducting treatment beyond two weeks [4]. Chronic ruptures are difficult to treat and frequently require reconstruction. There are different surgical techniques for reconstruction, using autografts, allografts, and synthetic materials [5-8]. Despite the description of several surgical procedures, there is no definitive "gold standard" in treating these injuries.

We report a case of neglected rupture of the patellar tendon and tibial fracture, which was treated with reconstruction with a single semitendinosus tendon, along with native tendon repair and McLaughlin augmentation of the reconstruction. Some benefits of our approach are that it doesn't require implants and is very simple, affordable, and efficient.

We obtained the patient's informed written consent and permission to publish all clinical data and diagnostic imaging in a medical setting.

## Case presentation

A 23-year-old patient presented at our outpatient clinic and complained of pain in the injured leg, inability to "lock" the knee in extension, and frequent falls, especially while walking down the stairs and downhill. Seven months before our visit, he suffered a gunshot wound (GSW) to his right knee in a mass shooting. Along with the other victims, he was transferred to a nearby hospital, where the on-call staff performed GSW irrigation and debridement in addition to external fixation, which bypassed the knee. After the postoperative radiograph was taken, external fixation was removed, the GSW was sutured, and he was discharged from the hospital.

**Figure 1:**
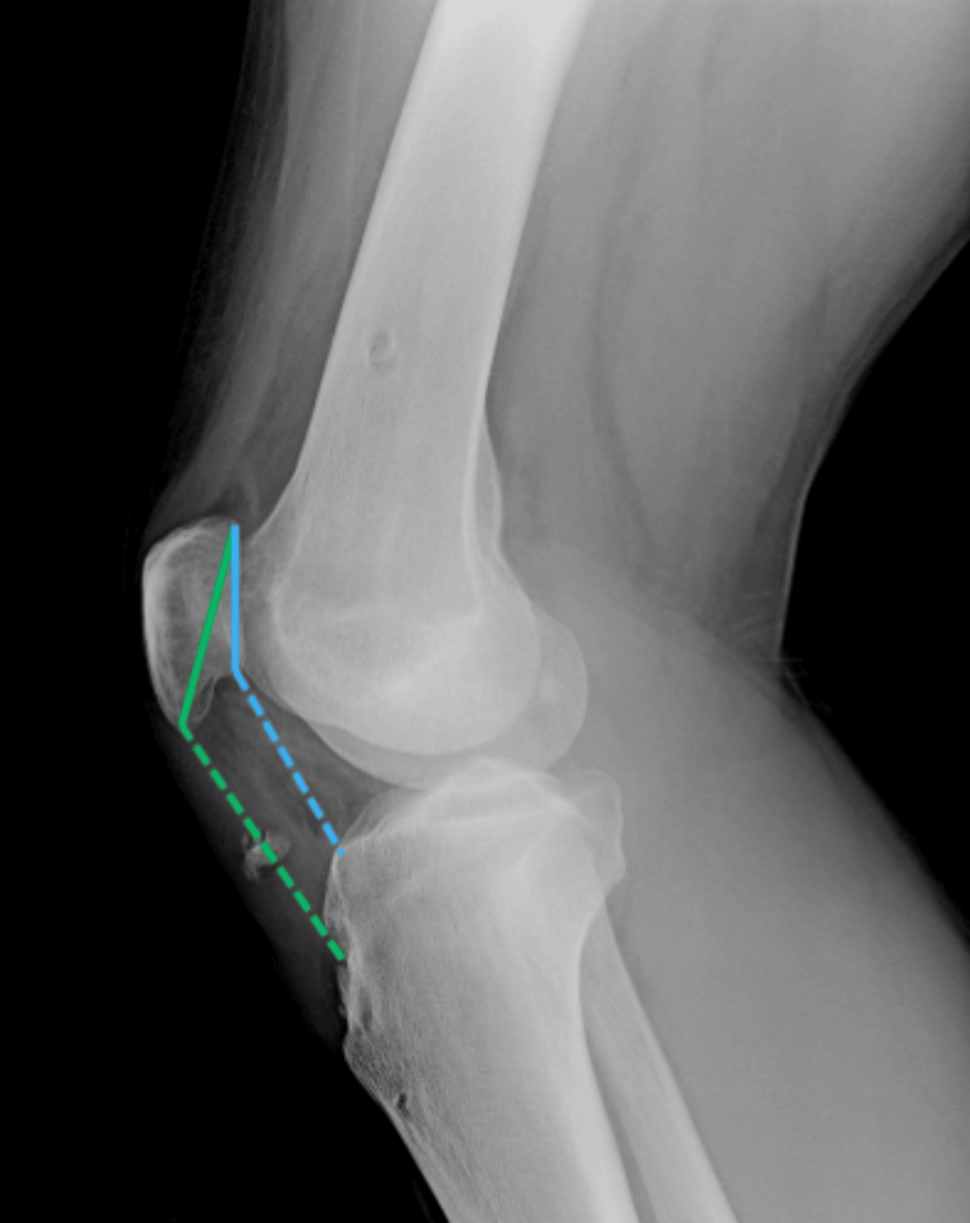
A bony fragment in projection of the patellar tendon and Insall-Salvati ratio (green line) and Caton-Deschamps index (blue line)

A thorough physical exam revealed no joint effusion, a horizontal scar in the projection of the patellar tendon, a palpable gap in the tendon's midline approximately 10 mm proximal to the tibial tubercle, and proximal translation of the patella. The patient was able to actively elevate the extended leg, but with a 15° extension lag of the knee. Other clinical findings regarding ligament stability and meniscal pathology were within normal limits, with no signs of neurovascular injury or acute inflammation. The radiological examination revealed a bony fragment in a projection of the patellar tendon and patella alta, with the Insall-Salvati ratio and the Caton-Deschamps index calculated at 1.35 and 1.50, respectively (Figure 1) [9].

**Figure 2:**
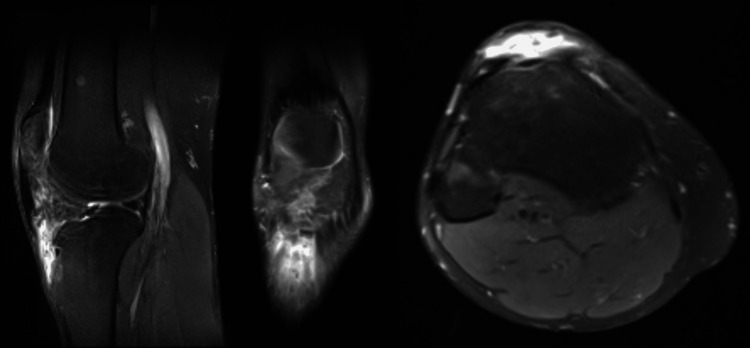
MRI of the injured knee (left to right: sagittal, coronal, transversal view)

The MRI exam revealed mid- to distal intrasubstance destruction of the patellar tendon tissue (Figure 2).

Surgical technique

**Figure 3:**
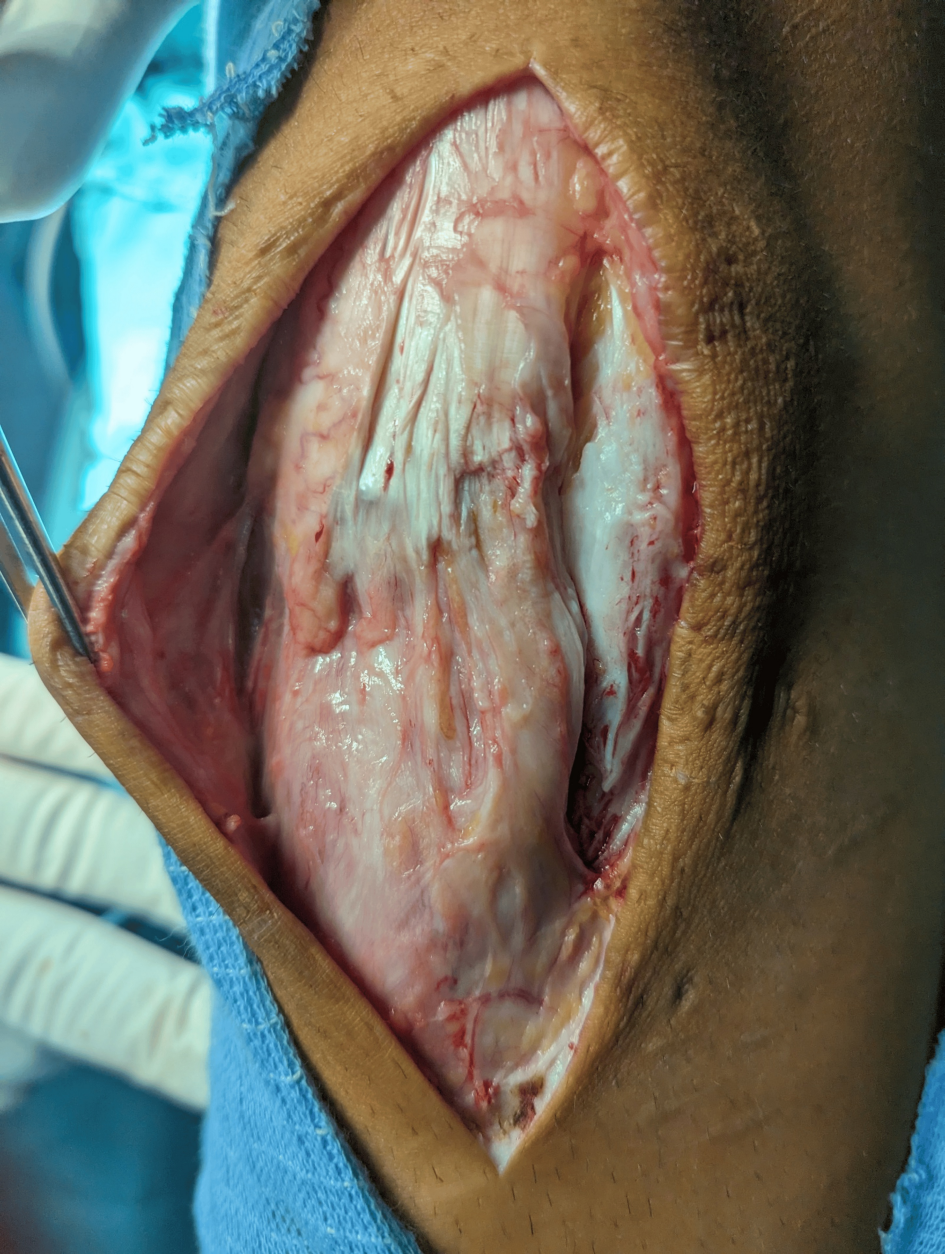
Surgical approach and exposed patellar tendon

The patient was placed in a supine position, general anesthesia was induced, and a sterile, single-use tourniquet (HemaClear®, OHK Medical Devices, Inc., USA) was placed on the upper thigh. A longitudinal incision was made over the earlier scar with the distal extension, exposing the tendon (Figure 3).

**Figure 4:**
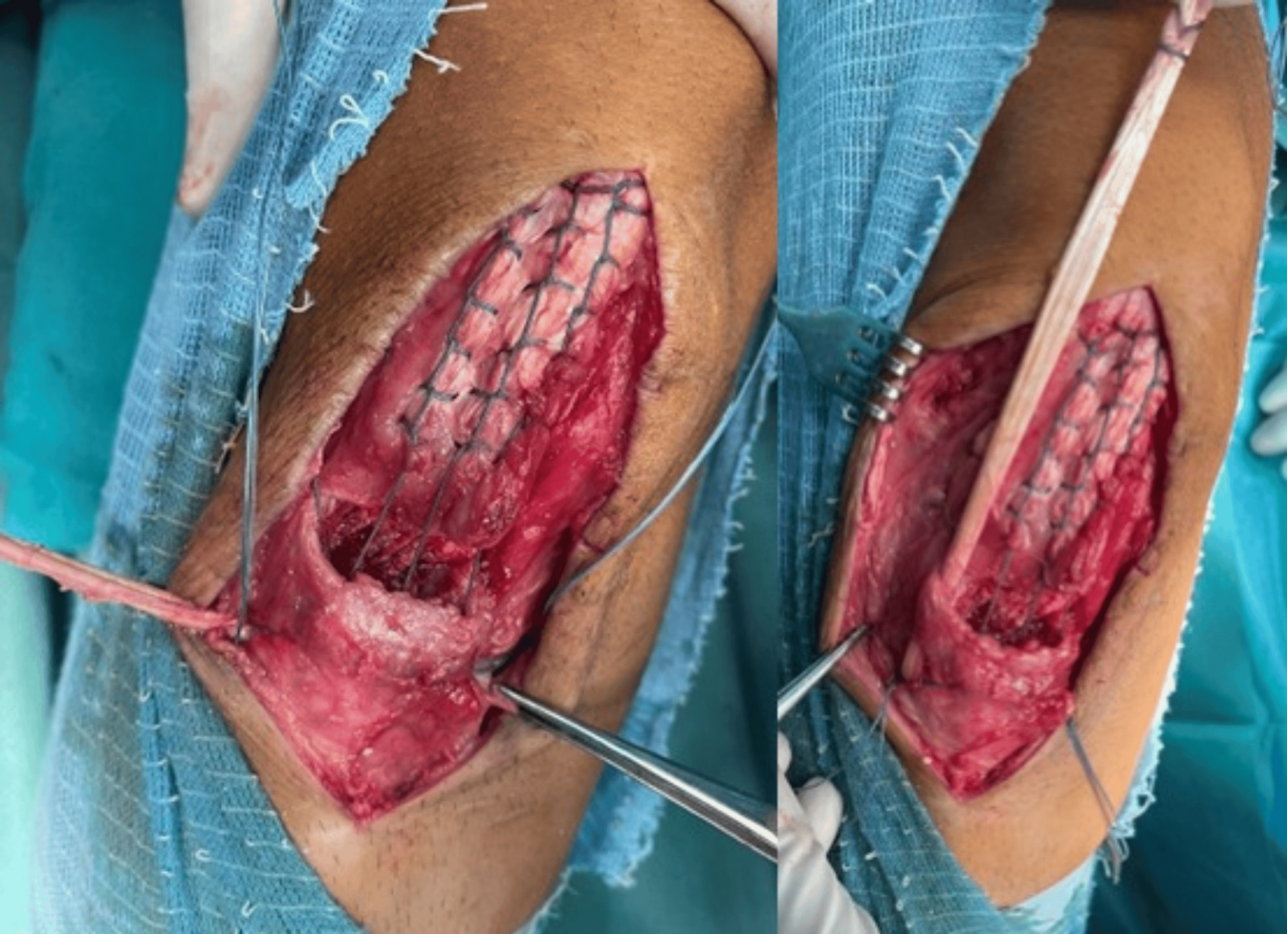
Pulling the graft through the tibial tunnel (left), then parallel to the patellar tendon (right)

Samples of scar tissue and a cotton swab were sent for microbiologic analysis, which came back negative. Excessive scar tissue was marked and debrided along with the bone fragment. The semitendinosus tendon was identified and stripped with an open striper, leaving distal insertion intact. The muscle was removed, and the free end was whipstitched with FiberLoop® (Arthrex, Inc., Germany). The native patellar tendon was debrided of scar tissue and stitched in the Krakow technique with #2 FiberWire® (Arthrex, Inc., Germany). The patellar tendon insertion site was identified, debrided, and curetted, and the healthy bone cradle was exposed. With the fluoroscopic control, one guide pin was placed in the projection of the tibial tubercle and the other through the midline of the patella. After the correct position was determined, tunnels were over-drilled with a 6mm reamer. The graft was pulled from medial to lateral through the tunnel, then parallel to the patellar tendon to the patellar tunnel, and then back on the medial side, parallel to the tendon to its insertion (Figure 4).

**Figure 5:**
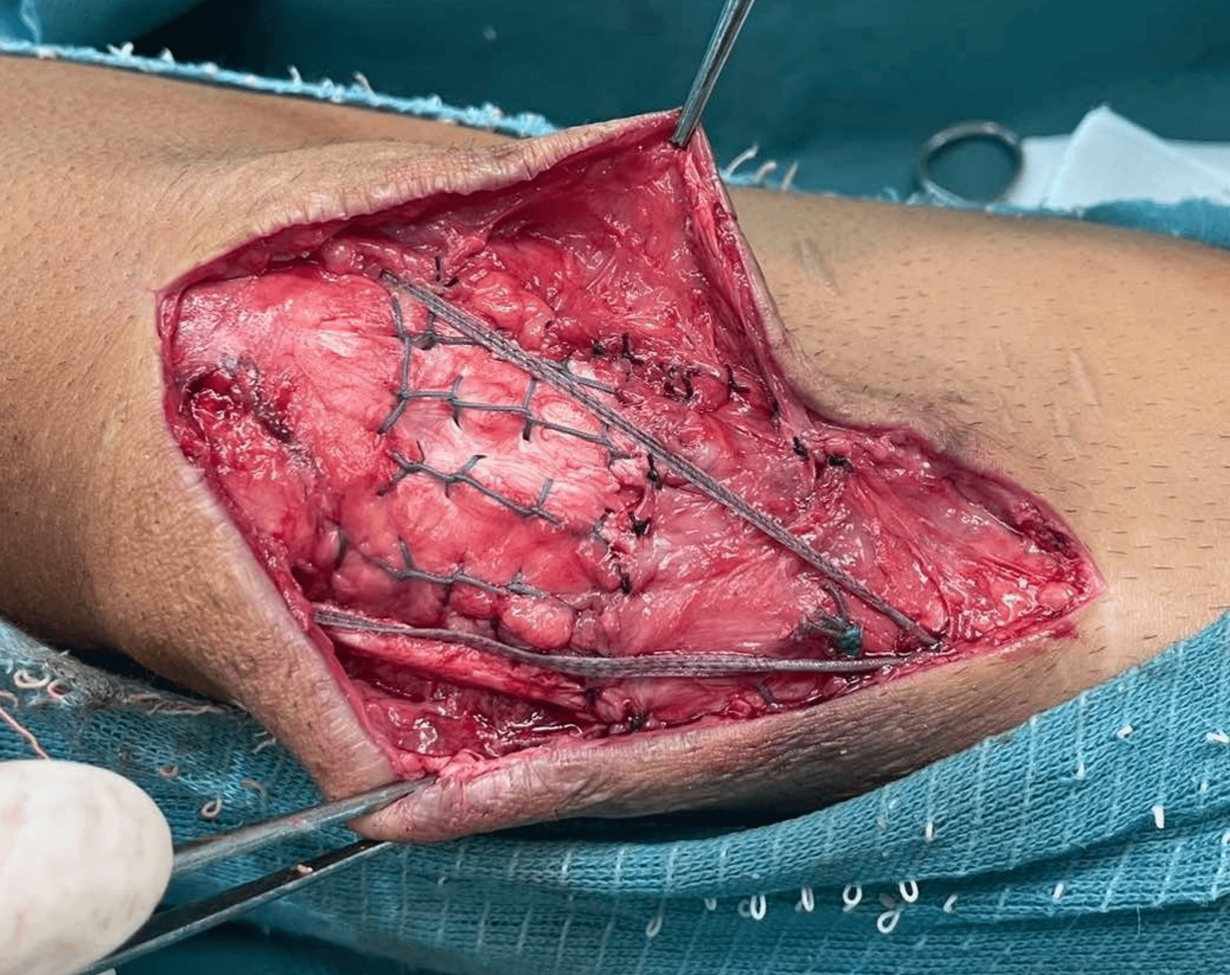
Final reconstruction of the patellar tendon

After reducing the patella to its height, defined with the intersection of its lower pole with the intercondylar notch roof, the graft was sutured to its insertion and surrounding soft tissue with a non-absorbable suture. By leaving enough scar tissue to reach the bone cradle, we were able to avoid the native tendon tissue defect. We then used the #2 FiberWire® to reinsert it into the bone cradle, which was then secured together through the tunnel. The McLaughlin augmentation with 2mm FiberTape® (Arthrex, Inc., Germany) was put in through the patellar tunnel while the knee was bent at 70°. It was then secured on the side of the tibial tubercle with a PushLock® (Arthrex, Inc., Germany) anchor. When the knee was moved to 90° of flexion, the final reconstruction was assessed, and no additional graft tension was observed. Reconstruction of the surrounding soft tissue was performed using absorbable sutures (Figure 5).

The wound was irrigated and sutured in a standard manner; the knee was placed in a tutor cast in full extension.

Postoperative period

On the first postoperative day, a knee brace was applied and locked in extension. Early physical treatment started by protocol with the objectives of reducing edema, enhancing lower limb musculature, and correcting the patient's gait. During the first two weeks, we took care of the wound and pain control. The patient commenced an isometric workout for the quadriceps and hamstrings, alongside strengthening of the contralateral leg muscles. We allowed walking with the crutches with partial weight bearing while the brace was secured at a 30° flexion angle. After two weeks, full weight bearing was permitted, and we initiated a weekly increase in flexion angle of 15°, achieving 90° by the sixth week, supplemented with gentle patellar mobilization. For the subsequent phase, the patient had to achieve a range of motion in the brace, without any swelling or joint effusion, and demonstrate an improvement in muscular strength compared to the beginning of physical treatment. Over the next six weeks, we completely released the brace; however, the patient stayed immobilized and maintained quadriceps and hamstring strengthening exercises with active range of motion activities. Commencing with the eighth week, we allowed for straight leg raises and stationary cycling without any resistance. The brace was removed after 12 weeks; the patient progressed with ipsilateral quadriceps strength as well as proprioception and balance exercises on the operated leg. Running was permitted after 24 weeks, whereas jumping and return to sport were allowed only after achieving 90% muscle strength of the contralateral leg and not before six months.

Results

**Figure 6:**
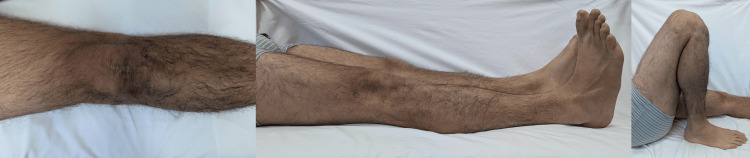
Aesthetic result and range of motion of the knee on a one-year follow-up

The postoperative period progressed without issues; the patient finished a comprehensive physical treatment program and returned to his usual daily activities. On a one-year follow-up, the patient was painless with a stable gait, active knee extension without the extension lag, and full range of motion of the knee (0-130°) with an aesthetically pleasing result (Figure 6).

**Figure 7:**
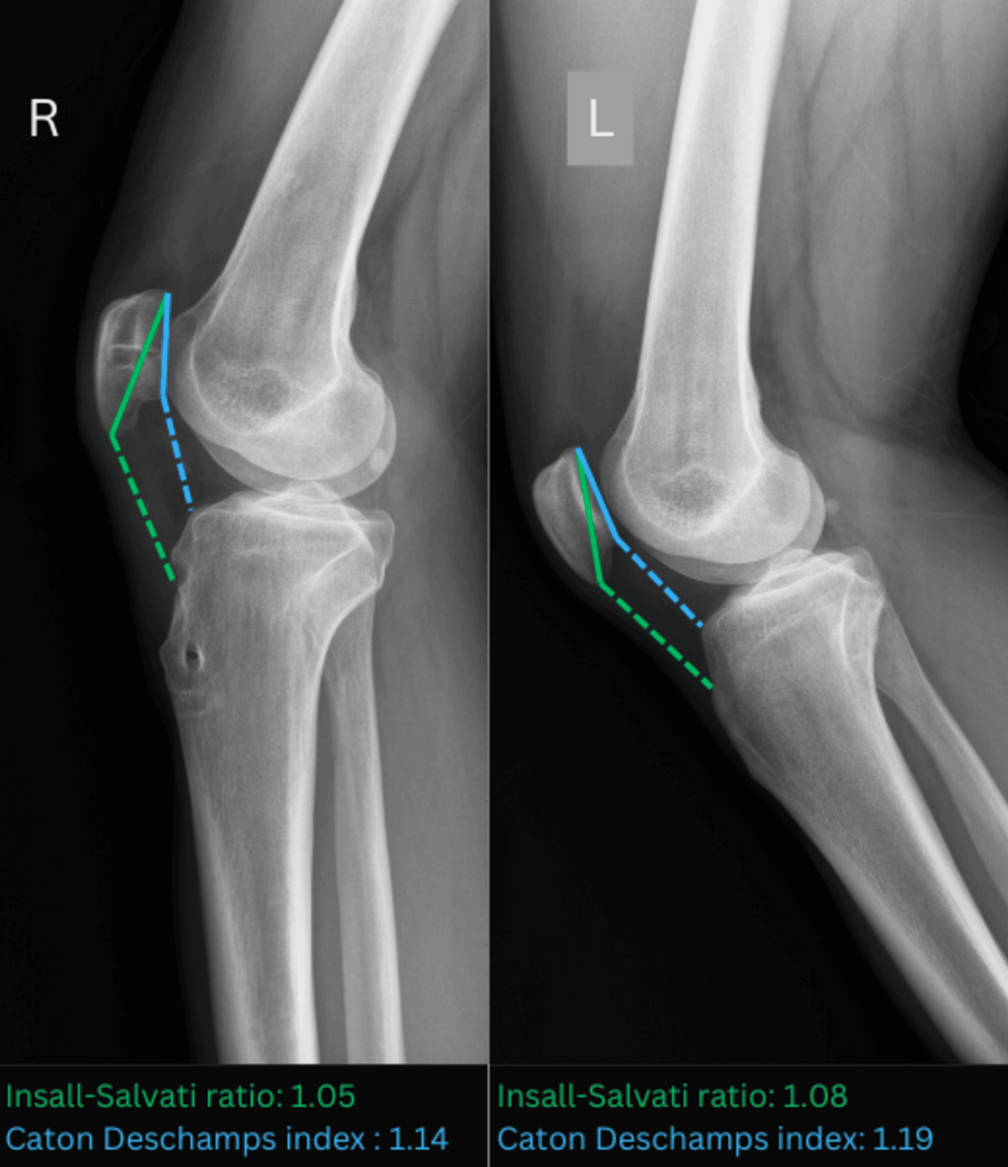
Comparison of the Insall-Salvati ratio (green line) and Caton-Deschamps index (blue line) of the injured (left) and uninjured (right) leg

The Insall-Salvati ratio and Caton-Deschamps index were calculated radiographically and compared to the uninjured side (Figure 7).

**Figure 8:**
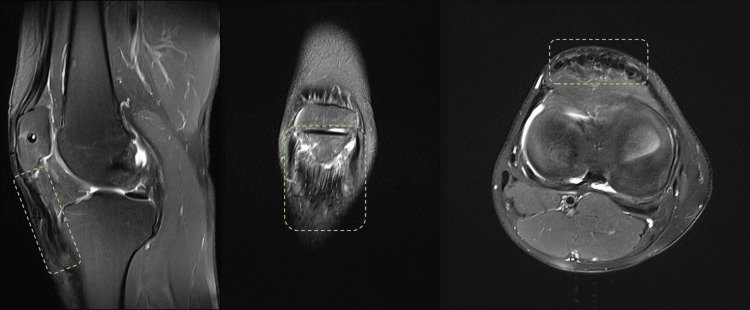
One-year follow-up MRI (green dashed line indicating full integration of the reconstruction)

The graft tissue had fully integrated, according to the MRI study conducted at a one-year follow-up (Figure 8).

## Discussion

Spontaneous tendon rupture is more common in patients with diabetes mellitus, chronic renal failure, and systemic connective tissue disorder. Our patient had no comorbidities, and the trauma (GSW) caused the injury. According to published data, the most common traumatic mechanism of injury was sports trauma, with forceful contraction of the quadriceps muscle [3]. Primary tendon repair is indicated in acute cases, while the tendon is not retracted, atrophied, and with quality tissue. Treatment of chronic cases necessitates reconstruction, with the usage of autografts, allografts, and synthetic materials.

Allografts after cadaveric striping need to go through the process of radiation or chemical processing, which changes the characteristics of the tissue, making them weaker and prone to rupture and reconstruction failure [10]. The third generation of synthetic material that is currently used in anterior cruciate ligament (ACL) reconstruction is LARS® (ligament augmentation and reconstruction system, LARS, surgical implants and devices, Arc-sur-Tille, France). Gao et al. conducted a multicentric study and showed good functional results and a low complication level in acute and chronic ACL ruptures [11]. Both allografts and synthetic material grafts are not available in our country.

However, there are a variety of autorafts available, such as hamstring tendon graft, contralateral bone-patellar tendon-bone (BTB) graft, Achilles tendon graft, and quadriceps tendon turndown [12]. Kim et al. conducted a recent systematic review, which found no difference in functional outcome and strength between hamstring tendon, BTB, or Achilles tendon grafts [13]. Overlooked patellar tendon ruptures are rare injuries without a large patient series follow-up in the literature.

Jarvela et al. used a semitendinosus-gracilis (STG) graft for reconstruction of the patellar tendon, fixed with an interference screw and staple, sacrificing the attachment and using a graft as free [14]. Cadambi et al. preserved the distal insertion of the STG graft, but they did not create a tibial tunnel with a graft-free end sutured to its insertion or to the proximal tibial periosteum after it passed through the tunnel in the patella [5]. Kim et al. suggest that preserving the tibial insertion of the hamstring tendon in ACL reconstruction results in a more viable graft and stronger distal fixation [15]. Using an animal model, Papachristou et al. compared ACL reconstruction with STG graft with and without maintaining tibial insertion, concluding that harvesting the graft while preserving tibial attachment could preserve a sufficient blood supply [16]. We retained the semitendinosus tibial insertion, believing it would provide additional stability and promote tendon-bone healing. Also, we preserved the gracilis muscle and its tendon, which can add to the knee motion in postoperative rehabilitation. We found that using a single tendon was sufficient for the reconstruction strength.

FiberTape® McLaughlin was used to augment the reconstruction, transmitting the force from the patella to the tibial tubercle, relieving the pressure from the graft and native tendon repair, and aiding in healing. Additionally, augmentation strengthens the construct, allowing for early rehabilitation and weight bearing. Some authors have used wire for the same purpose, but we avoid it due to the need for additional surgery to remove it and the inability to perform a control MRI of the knee. Interference screws or cortical fixation with uni- or bicortical buttons can be used for graft fixation [17-19]. We fixed the graft near its insertion with nonabsorbable sutures.

Potential complications include wound complications, postoperative infection, patella fracture, and tibial tubercle fracture when the transosseous tunnels are made.

## Conclusions

A patellar tendon rupture must be ruled out in every patient with a knee injury. Overlooked ruptures are difficult to manage because of ligament retraction and surrounding tissue scarring. Our case report demonstrates that in a neglected injury, one-stage reconstruction with a single semitendinosus tendon autograft can result in a favorable outcome. We believe that the technique mentioned above provides a solid construct that enables the restoration of knee function. There is no requirement for expensive implants, and the procedure is quite simple and repeatable.

Additionally, larger sample sizes and longer-term studies are needed to validate the effectiveness of this one-stage reconstruction technique. Further investigation into potential complications, such as re-rupture rate or potential patellar tracking issues, and comparisons with other surgical approaches would also be beneficial in determining the overall success rate of this procedure.
